# Low-Molecular-Weight Gelators as Base Materials for Ointments

**DOI:** 10.3390/gels2020013

**Published:** 2016-04-01

**Authors:** Yutaka Ohsedo

**Affiliations:** 1 Advanced Materials Research Laboratory, Collaborative Research Division, Art, Science and Technology Center for Cooperative Research, Kyushu University, 6-1 Kasuga-koen, Kasuga-city, Fukuoka 816-8580, Japan; ohsedo@astec.kyushu-u.ac.jp; Tel.: +81-92-593-1389; 2 Comprehensive Research Organization, Fukuoka Institute of Technology, 3-30-1, Wajiro-Higashi, Higashi-ku, Fukuoka 811-0295, Japan

**Keywords:** low-molecular-weight gelators, molecular gels, ointment, alkylhydrazide, alkylamide

## Abstract

Ointments have been widely used as an efficient means of transdermal drug application for centuries. In order to create ointments suitable for various new medicinal drugs, the creation of ointment base materials, such as gels, has attracted much research attention in this decade. On the other hand, the chemical tuning of low-molecular-weight gelators (LMWGs) has been increasingly studied for two decades because LMWGs can be tailored for different purposes by molecular design and modification. In this review, several series of studies related to the creation of ointment base materials with enhanced properties using existing and newly-created LMWGs are summarized.

## 1. Introduction

**Figure 1:**
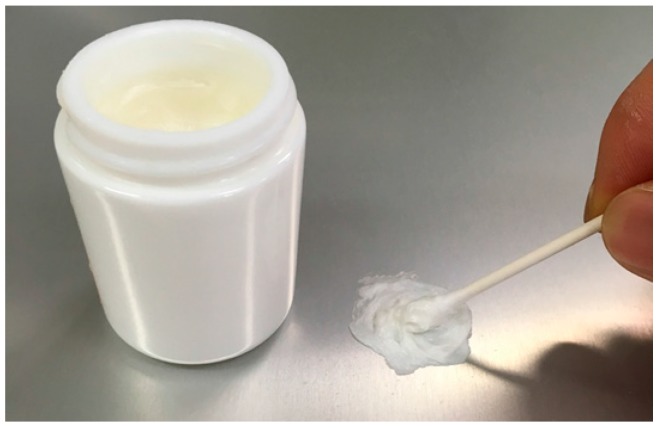
Photo of an ointment.

Ointments are used as a method for delivering medicinal agents by direct application to the affected area of the skin or by application to the surface of the skin near the affected area, depending upon whether the wound or condition is internal or external. This is termed transdermal drug delivery (Figure 1) [1,2]. It is interesting that many medicinal drugs are delivered to an affected area of the skin or to the vascular system through the skin by simple application of an ointment. Treatments using ointments do not require surgical excision and do not cause the external wound associated with, for example, injections. Consequently, the use of ointments is regarded as a relatively mild, non-invasive treatment method. On the other hand, although ointments can sometimes cause side effects, such as inflammation or irritation of the skin, these side effects are usually less severe than those associated with other treatment methods and are usually caused by the drug being applied and not the method of application. At present, ointments, which have been used since a long time, have become generalized not only as mild drugs and remedies but also as commodities, such as cosmetic creams and sunscreens in our daily life.

An ointment is composed of a medicinal agent and a base material that together form and maintain the structure of an ointment jelly. Thus, a base material must exhibit chemical stability and consistency of appearance in the presence of the formulated medicinal agent [3]. It must also cause low skin irritation and hypersensitivity and no discomfort to the patient [3]. Gels satisfy these requirements and are easy to prepare and formulate. Furthermore, they are suitable for use as ointment base materials because lipophilic medicinal agents easily dissolve in gels composed of oil; gels are flexible, spreadable, and not sticky; they can be applied to hair-covered areas; and they exhibit good adhesion to the skin, promoting effective delivery of medicinal agents to the affected area [4].

While the use of polymer gels is well-known and thoroughly investigated, molecular gels, which are constructed by the self-assembly of low-molecular-weight gelators (LMWGs), have attracted much attention as a new soft materials in the past two decades [5,6,7,8,9,10,11,12,13,14,15,16,17,18]. As discussed below, many old and well-known ointment base materials are now recognized as molecular oil gels. However, the properties of molecular gels can be molecularly designed and tuned, and these advantages make molecular gels historically proven and advanced soft materials.

In this review, the author presents a summary of studies on the application of molecular gels (mainly organogels for oil gels) used as base materials for ointments. These gels exhibit unique properties because they are composed of LMWGs; however, they exhibit some similar properties to polymer gels because of the formation of networks of fibers composed of LMWGs. First, ordinary ointments and molecular gels with thixotropy, which is a required property for ointment applications, are discussed. Then, recent studies on several organogelators for ointments are summarized. Finally, recent developments of new low-molecular-weight organogelators for ointments are described with respect to their required mechanical properties, including thixotropy.

It should be noted that application studies of molecular hydrogels and organogels for drug delivery have been summarized in the literature [19,20,21,22]. On the other hand, while studies on injectable molecular hydrogels are useful for understanding the design and tuning of the rheometric properties of molecular gels in an ointment, these studies are not covered in this review. The studies of injectable molecular hydrogels have been well summarized in a review by Bing Xu [23].

## 2. Ointments

There are many representative medicinal drugs applied as an ointment to the skin as topical agents, such as anti-inflammatory agents (e.g., methyl salicylate), local anesthetics (e.g., lidocaine), immunosuppressive agents (e.g., glucocorticoids), and vasodilators (e.g., isosorbide mononitrate) [1,2]. Low toxicity and high stability to the drugs contained are required in case of effective ointment base materials.

Ointments can be used as the active ingredient in dermal and transdermal patches. Owing to the development of many types of patches, ointments will become increasingly applied in this form. According to the wide range of future uses of ointments and the potential demand for ointment base materials with improved properties suitable to various types of medicinal drugs, the re-examination of both traditional and newly-developed base materials has been performed. Generally, ointments are produced by mixing and dissolving a medicinal agent, a base material, and a non-volatile oil. Typical non-volatile oils used in ointments are edible fats and oils, such as vegetable oil and animal fat, and synthetic oils and esters, such as silicones and isopropyl myristate, which are generally used in cosmetics. These fats and oils must be safe for human use.

Franz-type vertical diffusion cells are generally used to obtain data on the transdermal absorption of ointments *in vitro*. In this apparatus, an ointment containing a medicinal drug is placed on an appropriate membrane, e.g., a cellulose acetate membrane or pig skin as an analog of human skin, and a buffer or aqueous solution near body temperature is used as a model for a physiological aqueous solution for receiving the permeated or diffused medicinal drug from the ointment through the membrane [24,25]. Controlled drug delivery with sustained release is required for ointments, and their suitability is evaluated from the transdermal absorption data for the ointment by the use of theoretically-developed models [26,27,28,29].

Liposomes, microgels, and LMWGs have been examined as newly-developed base materials [30]. On the other hand, Dayan classified ointments according to their material forms into four systems: liposomes, elastic vehicles, particulate systems, and molecular systems (dendrimers) [2]. Taking into consideration both new and well-known materials, these base materials can be reclassified into the following categories: liposomes, polymer gels as elastic vehicles (including natural and synthetic polymer gels), particulate systems, and molecular systems (including dendrimers and molecular gels). Of the newly-developed base materials, molecular systems have some advantages, such as well-defined molecular structures (compared with polymeric compounds, which are usually a mixture of polymers with different chain lengths), the potential for molecular design because of their well-defined molecular structure, and easy tuning by chemical modification of their well-defined structures. In particular, LMWGs are newly-understood gel-forming molecular agents; they are already used as base materials for some traditional ointments, e.g., petroleum jelly and hydrocarbon-based ointments, such as Vaseline®. Thus, LMWGs are a unique type of base materials for ointments with tunable molecular properties. There is much of room for improvement in their properties by molecular design.

## 3. Molecular Gels

**Figure 2:**
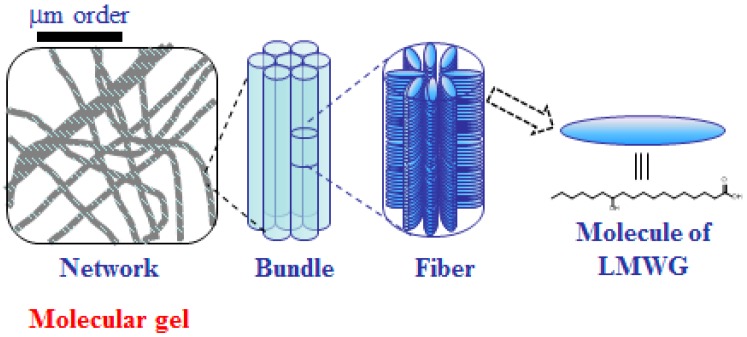
Schematic illustration of the formation of molecular gel.

Molecular gels are defined as crystalline fiber networks constructed from LMWGs in self-assembled manner that incorporate solvents within the networks, resulting in a gel-like appearance, much like that of polymer gels (Figure 2) [5,6,7,8,9,10,11,12,13,14,15,16,17,18]. When an aqueous solution is used as the solvent for a molecular gel, the gel is termed a molecular hydrogel, and when an organic solvent is used as the solvent, the gel is termed a molecular organogel. In general, LMWGs can be dissolved in hot organic solvents or water, and the solutions give molecular gels on cooling to room temperature. The state of a gel is initially verified with the naked eye by observing its formation and is then quantitatively evaluated by the use of rheometric equipment.

Using molecular gels as base materials for ointments is one of the most promising applications of LMWGs as soft materials. Molecular gels composed of LMWGs for use in ointment usage have several advantages, such as easy preparation and the possibility of improving their properties compared with those of polymeric compounds by molecular design. Preparation of LMWGs can be conducted inexpensively, with commercially available ingredients and a practical synthesis that results in a product suitable for use as an ointment. In general, LMWGs are easy to synthesize and prepare because of their low molecular weights and well-defined structures.

Non-toxicity to humans is absolutely necessary. The design of non-toxic LMWGs that retain their required properties can be attained by tuning their chemical structure precisely using the results of toxicity tests. In terms of their molecular properties, molecular gels have poor mechanical strength, low durability, and poor long-term stability of appearance compared with polymer gels, which contain polymer networks resulting from multiple interactions between polymer chains. Despite the inferior properties of molecular gels compared with polymer gels, good stability and thixotropic behavior can be observed in molecular gels composed of molecularly designed LMWGs.

Thixotropy is defined as mechanically induced reversible sol-to-gel or gel-to-sol changes and is generally observed in polymeric or inorganic nanosheet solution systems [31,32,33,34]. This type of behavior has been observed in some molecular organogels and hydrogels [35,36,37,38,39,40,41,42,43,44,45,46,47,48,49]. Thixotropy is an essential material property for applying, spreading, and expanding the material while retaining its appearance and form. Consequently, thixotropy has received much attention as a required property of base materials for ointments. When applied to skin patches, the poor mechanical strength of molecular gels will be compensated by the supporting patch substrate. However, molecular gels with thixotropy have a wider range of applications as base materials for medicinal drugs from patches to ointments. The thixotropic behavior of gels is initially assessed with the naked eye by observing its recovery from the broken state on applying mechanical force and then quantitatively evaluated by the use of rheometric equipment [31,32,33,34,35].

Since medicinal drug molecules commonly have lipophilic properties, molecular organogels, prepared from low-molecular-weight organogelators, are used to retain the medicinal molecules within a gel network, and molecular hydrogels, prepared from low-molecular-weight hydrogelators, are used to support micelles containing the medicinal molecules within a gel network. In the case of ointments composed of molecular gels, there is a tendency to use the latter micelle-dispersed hydrogels owing to the frequent presence of aromatic rings in organogelators, leading to their use being avoided by medical personnel [22]. Taking this into account, the author introduces LMWGs without aromatic rings in their chemical structures for ointment usage. In the following sections, the author summarizes the studies on existing and newly created LMWGs as new candidates for ointment base materials.

## 4. Studies Using Existing LMWGs

**Figure 3:**
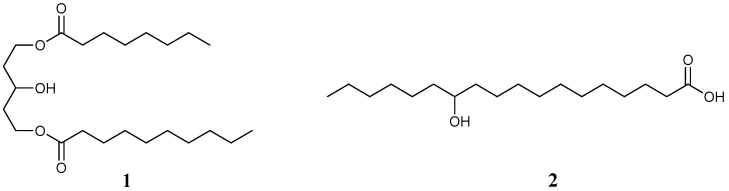
Chemical structures of existing low-molecular-weight gelators (LMWGs).

Glycerol esters, which are compounds commonly found in vegetable oils and animal fats, have been used as LMWGs for oils. The resulting organogels have similar feeling on skin application as soft paraffin (petroleum jelly), which is a mixture of hydrocarbons that forms a gel-like material. As an organogelator for use in ointments, Miglyol 812® (caprylic/capric triglyceride, Sasol Ltd. Johannesburg, South Africa, Figure 3(**1**)) has been used by Pénzesa *et al. in vitro* and *in vivo* (rats) in the molecular gel state with the anti-inflammatory drug piroxicam, with white petrolatum and liquid paraffin being used as references [50]. This study showed that increasing the concentration of piroxicam led to the inhibition of edema, subject to a power law correlation, and that the molecular organogel could be used as an ointment base material with medicinal drugs *in vitro* and *in vivo* more effectively than the reference materials.

The natural glycerol ester derivative lecithin has also been used as LMWG for oils and is a candidate for an ointment base material. Pal *et al.* showed that a soy lecithin-based organogel with a sunflower oil-water mixture and the antibiotic metronidazole functioned as an ointment with controlled release *in vitro* [51]. In this study, the rheometric properties of the organogels were measured, and their thixotropic shear-thinning behavior indicated that they may be easily spreadable and, hence, used for topical application.

12-Hydroxystearic acid (12-HAS, Figures 3(**2**)) is a fatty acid derivative well-known for its ability to form molecular organogels. 12-HSA functions as LMWG for vegetable oils and is often used as an additive to assist in the disposal of cooking oils. 12-HSA is a candidate for an ointment base material, and its use in oral controlled-release organogels has been studied. Iwanaga *et al.* reported that an organogel composed of 12-HSA, soybean oil, and the lipophilic medicinal drug ibuprofen formed a controlled drug release material *in vitro* and *in vivo* [52]. Remarkably, they also showed that organogels composed of 12-HSA and soybean oil with a hydrophilic drug such as ofloxacin, which is sparingly soluble in soybean oil but at a sufficient level for drug efficacy, formed a controlled drug release material *in vitro* and *in vivo* [53]. These results could confirm a potential use of 12-HSA for ointment.

## 5. Studies Using New LMWGs

**Figure 4:**
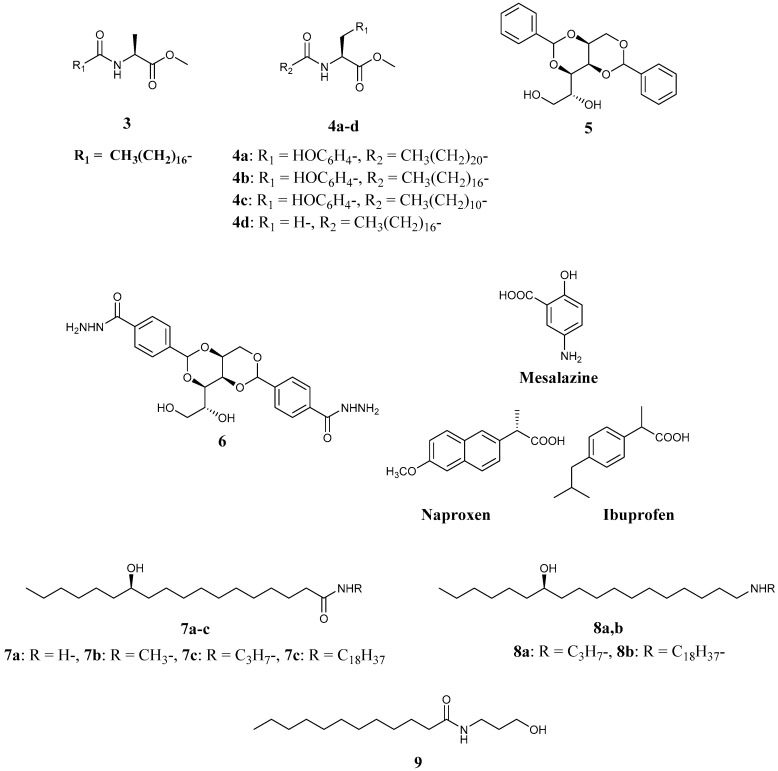
Chemical structures of new LMWGs.

Amino acid derivatives have been studied as a promising family of LMWGs. Using amino acid derivatives, Leroux *et al.* studied organogel systems with the ability to be used as implants, and their results are suggestive and informative for the study of ointment creation using gelators (Figure 4(**3**,**4a**–**d**)). They studied organogels composed of LMWGs containing a tyrosine unit and the parasympathomimetic drug rivastigmine and found that the drug was released slower in stiff organogels with higher Young’s moduli, suggesting the importance of evaluating the mechanical properties of molecular gels for drug delivery and ointments [54,55]. Leroux *et al.* reported that the difference between the results observed in *in vivo* and *in vitro* experiments, which were inferior *in vitro*, was ascribable to the existence of *in vivo* enzymes, which led to enzyme-related degradation and erosion of the gel matrix, thereby affecting the diffusion mechanism of the drug [55]. Consequently, they created molecular organogels composed of sunflower oil, a lipase (which is often present in humans as esterase), and the antibiotic ceftiofur sodium. They reported that the lipase-containing organogels worked as controlled drug-release materials *in vitro* with the same performance as that *in vivo*. This strategy established a methodology for the fast creation of drug delivery materials *in vitro* without the need to change the gel formulation responsible for inferior results obtained *in vivo*. These results, and the concept developed, are important for the design of ointments using LMWGs because they show that the effect of biomolecules such as enzymes on the efficacy of ointments needs to be considered.

1,3:2,4-Dibenzylidene-d-sorbitol (DBS, Figure 4(**5**)) is an old type of LMWG for organic solvents that forms molecular organogels known for over 100 years, and it has been used as an additive in cosmetics and dental materials [56]. In recent years, molecularly designed and modified DBS derivatives have been created as new LMWGs with improved properties [56]. Smith *et al.* have created novel DBS derivatives containing hydrazide units (DBS-CONHNH_2_, Figure 4(**6**)) and examined two-component hybrid molecular hydrogel formations composed of DBS-CONHNH_2_ and medicinal drugs, such as mesalazine, naproxen, and ibuprofen (Figure 4), which exhibited pH-controlled drug release *in vitro* [57]. Furthermore, the stiffness of the hybrid hydrogels revealed that they exhibited different stiffness depending on the lipophilicity or hydrophilicity of the incorporated drug, with lipophilic drugs forming a hard gel and hydrophilic drugs forming a soft gel. Although the materials studied were not ointments, it is interesting to note that the medicinal drug acted not only as a component of the fibers in the two-component hydrogel but also as a tuning additive to control gel stiffness. This new concept for creating molecular gels will be useful for the molecular design of ointment base materials as well as other drug release materials composed of molecular gels.

In recent years, 12-HSA has been considered as one of the most important core chemical structures for new high-performance organogelators. The relationship between the material properties of molecular gels composed of 12-HSA derivatives with different chemical structures was thoroughly studied [58,59,60]. They performed systematic studies on simple organogelators that may be applicable as ointment base materials owing to their thixotropic behavior and reported the formation of organogels composed of oils (silicone oil [58] and safflower oil high in oleic acid [59]) and a family of modified 12-HSA with an amide (Figure 4(**7a**–**c**,**8a**,**b**)). Related to this studies, the basic material properties involving thixotropic behavior of toluene organogel composed of simple gelator with an amide was also evaluated (Figure 4(**9**)) [60]. Their results showed the importance of basic research using simple and the possibility to chemical tuning of gelators to produce improved LMWGs as potential candidates for ointment base materials [13].

**Figure 5:**
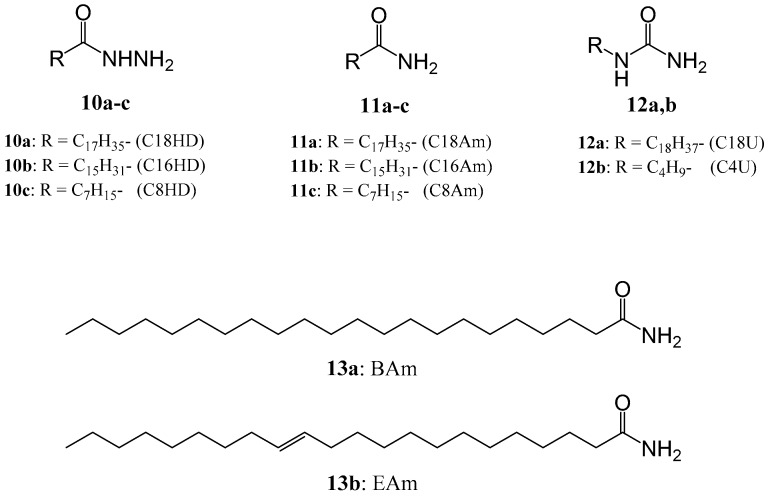
Chemical structures of LMWGs for mixed molecular gels.

Recently, the author’s group found that simple alkylhydrazide derivatives (Figure 5(**10a**–**c**)), stearohydrazide (C18HD, **10a**), palmitohydrazide (C16HD, **10b**), and octanohydrazide (C8HD, **10c**), exhibit organogelation abilities with organic solvents having various dielectric constantsfrom 2 (*n*-hexane) to 66 (propylene carbonate) [61]. Previously, they have not been recognized as organogelators. Although alkyhydrazide molecular gels have poor mechanical properties, it was found that mixing alkylhydrazides with different alkyl chains enhance the mechanical properties of the alkylhydrazide molecular organogels [62]. Furthermore, the gelation ability of mixed gels (such as the critical gelation concentrations) are improved comparing with those of each single alkylhydrazide organogelator. In addition, the mixed alkylhydrazide molecular organogels showed thixotropic behavior, while single alkylhydrazide did not show thixotropic behavior [62]. Better thixotropic behavior become observed for gels containing increased C8HD, such as C18HD/C16HD/C8HD for 1/1/10 (w/w/w) in an organic solvent.

Although several molecular gels showed the mixing-induced enhancement of molecular gel properties [63,64,65], our results involved simple mixing of gelator homologues containing a hydrogen-bonding unit and different linear alkyl chains. Consequently, molecular organogels with enhanced gelation abilities and thixotropic behavior can be obtained by simply mixing commercially available alkylamide (octadecaneamide: C18Am, hexadecaneamide: C16Am, and octaneamide: C8Am, Figure 5(**11a**–**c**)) [66] and alkylurea organogelators (octadecylurea: C18U and butylurea: C4U, Figure 5(**12a**,**b**)) [67], respectively.

To observe microstructures of mixed xerogels by scanning electron microscopy showed increase of network density by changes of shape of the network component from sheet-like or tape-like crystals to fiber-like crystals in increasing the ratio of gelator with shorter alkyl chain [62]. The increase of network density will have the contribution to the mixed molecular gels with improved mechanical properties involving thixotropic behavior. In mixed gels, it may be possible to crosslink the finer fibers composed of mainly C8HD with those of C18HD and C16HD resulting in enhancement of mixed gel properties. The detail of mechanism for enhancement of mixed molecular gels is currently under investigation. In addition, these types of gelation ability and thixotropic behavior enhancements were observed in molecular hydrogel systems by mixing hydrogelators with different alkyl chains [68], as well as by mixing hydrogelator and nanosheet [69], probably because of the enhancement of network quality described above.

Furthermore, multicomponent alkylamide organogels composed of C18Am, C16Am, and C8Am containing non-volatile oils, such as olive oil and squalane, were prepared for examining its properties of controlled drug release. Even in the presence of these non-volatile oils, a mixed alkylamide molecular gels showed thixotropic behavior. This organogel showed controlled drug (antipyrine) release in diffusion kinetics and will become one of candidates of spreadable host material for ointment base material [70].

**Figure 6:**
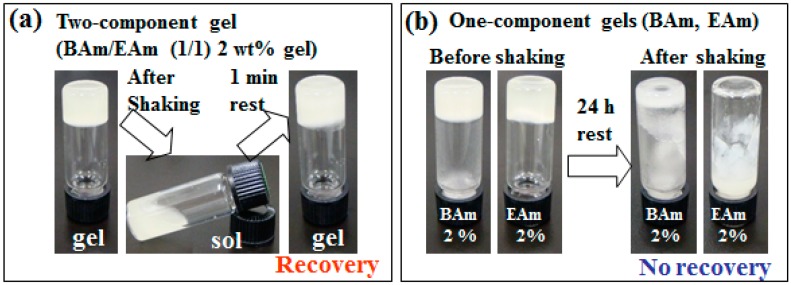
Photographs of thixotropic tests. (**a**) Two-component 2 wt % olive oil gel (BAm/EAm 1/1 (w/w)); and (**b**) one-component 2 wt % olive oil gels (BAm and EAm) [72]. Reproduced by permission of The Royal Society of Chemistry (RSC) on behalf of the Centre National de la Recherche Scientifique (CNRS) and the RSC.

Mixed alkylamides with long-chain and short-chain alkyl groups formed thixotropic gels even in non-volatile oils. However, thixotropic multicomponent alkylamide organogels containing non-volatile oils showed drug release ability, according to its MSDS data, C8Am will bring about eye and skin irritation [71]. Therefore, the author’s group used only the long-chain alkylamides behenamide (BAm, **13a**) and erucamide (EAm, **13b**) as organogelatos in order to create new thixotropic non-volatile oil gels for controlled drug release (Figure 5). These alkylamides are not known as LMWGs. The mixed oil gels demonstrated thixotropic behavior, while the corresponding one-component oil gels did not show such behavior (Figure 6). It was found that the incorporationof a drug (antipyrine) in the mixed olive oil gel enabled its slow and controlled drug release. While the olive oil solution of the drug released at 60 wt % of the drug after 7 h, 30 wt % of thedrug in a mixed gel (BAm/EAm, 1/1 (w/w)) olive oil gel was released in Fickian diffusion kinetics [72]. Efforts to tune the mixed gel properties and open up the option of incorporated drug for ointment base materials are underway.

## 6. Conclusions

In this review, the author has summarized several studies that aimed to create better molecular organogels applicable to ointment base materials by chemical approaches using LMWGs. The selection of suitable molecular gels for combination with new medicinal drugs and other additives needed to produce an ointment is challenging. Of course, the reexamination of existing molecular gels as ointment base materials is important; however, new molecular designs and new strategies for creating LMWGs that are suitable for ointments are required for the quick development of LMWGs for use with various medicinal drugs. The development of better treatments through the use of ointments will enhance our quality of life. Thus, more research in order to accumulate chemical libraries of different LMWGs with required properties such as thixotropy is required.
